# Early life environmental predictors of asthma age-of-onset

**DOI:** 10.1002/iid3.27

**Published:** 2014-07-26

**Authors:** Olivia R Ferry, David L Duffy, Manuel A R Ferreira

**Affiliations:** QIMR-Berghofer Medical Research InstituteBrisbane, Australia

**Keywords:** age, atopy, IL6R, infections, tobacco

## Abstract

Prevention strategies that delay the onset of asthma may improve clinical outcomes. To identify early life environmental exposures associated with asthma age-of-onset and potential genetic modifiers of these exposures, we studied 1085 subjects with physician-diagnosed asthma and disease onset at or after age two. Subjects reported retrospectively on their exposure to 17 environmental factors before the age of two. The presence of individual or combinations of these early life exposures was then tested for association with variation in asthma age-of-onset. For exposures significantly associated with age-of-onset, we tested if 26 single nucleotide polymorphisms (SNP) with an established association with allergic disease significantly modified the effect of the exposure. Five environmental exposures were significantly associated with variation in asthma age-of-onset after correction for multiple testing: carpet at home (*P* = 6 × 10^−5^), a serious chest illness (*P* = 10^−4^), father a cigarette smoker (*P* = 6 × 10^−4^) and direct exposure to father's smoking (*P* = 3 × 10^−4^). Individuals with early childhood asthma onset, between the ages of two and six, were 1.4-fold (CI 1.1–1.9) more likely to report having lived in a house with carpet and 2.1-fold (CI 1.3–3.5) more likely to report suffering a serious chest illness before the age of two, than asthmatics with later disease onset. We further found these individual risks to increase to 3.2-fold (CI 1.7–6.0) if carpet exposure and suffering a serious chest illness co-occurred before age two. Paternal smoking exposures were less likely to be reported by asthmatics with early when compared to later disease onset (OR 0.5, CI 0.3–0.7). There were no significant SNP interactions with these environmental exposures after correction for multiple testing. Our results suggest that disease onset in individuals at a high-risk of developing asthma can potentially be delayed by avoiding exposure to carpet at home and preventing serious chest illnesses during the first 2 years of life.

## Introduction

Asthma is an heterogeneous disease with epidemiological studies suggesting that it can be classified into a number of clinical phenotypes, based on age-of-onset, duration of disease, and clinical characteristics [1–3]. The clinical phenotype considered most severe is persistent atopic asthma, characterized by an early childhood onset of asthma, before the age of six, worsening lung function over time and no remission of disease in adulthood [3–5]. This persistent form of asthma has been linked to reduced asthma control leading to an increased number of emergency care visits [6]. Furthermore, studies analyzing the respiratory function of adult asthmatics have found that the longer the duration of asthma, the greater the decline in respiratory function [5,7–9]. Thus, there is significant potential to improve clinical outcomes for asthmatics if primary prevention strategies are identified that delay the onset of asthma.

The early life environment is considered to be critical in asthma pathogenesis due to the continued development of the respiratory and immune systems that occurs postnatally [10,11]. For example, in a study of individuals at a high risk of asthma, postnatal and not prenatal exposures were found to lead to immunological changes and an increased risk of allergic sensitization and asthma [12]. Additional studies have found that the first years of life are a critical period of increased vulnerability of the developing lungs [3,13]. Thus, early life exposures are considered to have a greater impact on asthma risk than exposures later in life [11,14].

To date, a large body of research has identified early life environmental risk factors for asthma such as viral respiratory illnesses, environmental tobacco smoke (ETS) exposure, household exposures to pets, moulds and dust, breastfeeding, and exposure to pollutants [13,15]. Despite these findings, conflicting directions of effect for particular risk factors have been reported. For instance, early life exposure to pets has been found to be protective in some studies [16] but not others [17,18]. On the other hand, in studies comparing non-asthmatics with early-onset asthmatics, environmental factors with the strongest association with disease risk include viral and bacterial respiratory infections, ETS exposure and early allergic sensitization [19–21].

In addition, the most effective primary prevention studies of asthma have involved the elimination of multiple environmental risk factors, indicating that an individual's risk of developing asthma is more realistically assessed if the exposure to combinations of environmental risk factors is considered [22]. Furthermore, because asthma is heritable [23], genetic variants also significantly contribute to an individual's asthma risk, either independently of, or by interacting with, environmental risk factors. For example, the identification of the first confirmed genetic risk factor for early-onset asthma on chromosome 17q12-21 [24] was followed by a number of studies that searched for significant interactions between the asthma risk variants in this region and exposure to specific early life environmental factors [25–27]. These studies found that the risk of disease conferred by a single nucleotide polymorphism (SNP) near the *ORMDL3* gene was significantly enhanced by both ETS exposure and respiratory infections [27,28].

To our knowledge, to date no studies have compared rates of exposure to environmental risk factors between individuals who develop asthma at a young age and those who develop asthma later in life. This analysis between two asthmatic groups is important as it enables the direct estimation of the effects of environmental risk factors on age-of-onset per se. In contrast, the method most commonly used in previous studies of comparing unaffected controls separately with either early-onset asthmatic cases or late-onset asthmatic cases is partially confounded by the effect of these risk factors on asthma incidence.

Therefore, the aims of this study were to (1) test the association between early life exposures and variation in asthma age-of-onset; and (2) test whether SNPs recently reported to associate with the risk of asthma or other allergic diseases through genome-wide association studies (GWAS) significantly modify the effect of environmental risk factors on asthma age-of-onset.

## Methods

### Participants

Subjects included in this study are a subset of a larger cohort of 4820 twins and relatives who participated in the Asthma and Allergy study carried out by the Queensland Institute of Medical Research (QIMR), as described in detail elsewhere [29]. For this study, we selected a subset of 1085 unrelated individuals who reported having previously been diagnosed with asthma by a doctor and who had an asthma age-of-onset at or after the age of two. Individuals who reported an age-of-onset between birth and age two were excluded in order to ensure that the environmental exposures selected for analysis (see below) preceded disease onset.

Of the 1,085 individuals, 682 (63%) were tested between 1992 and 1994 (Study 1), and 403 (37%) were tested between 1995 and 1998 (Study 2). Different ascertainment procedures were used for each study [29]. Briefly, Study 1 recruited families with at least one asthmatic twin, whereas Study 2 preferentially recruited families with two asthmatic twins or siblings. As a result, when compared to Study 1, asthmatics from Study 2 were more likely to report a family history of allergic disease (76% vs. 92%) and to suffer from other allergic diseases, for example, hay fever (55% vs. 77%).

### Assessment of environmental exposures

Participants (or their parents if under the age of 16) completed a questionnaire that retrospectively assessed asthma diagnosis and age-of-onset, early life environmental exposures and family history of asthma. In addition, 517 of these 1085 participants underwent clinical testing which included lung function and allergy testing. The questionnaires and the methods used for clinical testing are described in detail in Ferreira et al. [29]. From these questionnaires, we extracted responses for 17 questions concerning environmental factors the participant was exposed to before the age of two (Supplementary Table S1). These included questions about respiratory illnesses, otitis media, the location and type of house lived in, household exposures to pets and carpet, breastfeeding and both paternal and maternal smoking behaviors. There was also one question that assessed in-utero exposure to maternal smoking. Eight of the 17 questions were not included in Study 1, and so for these the analysis is based on a smaller sample size (up to *N* = 403).

### DNA genotyping and selection of SNPs

A subset of 318 asthmatics had previously been genotyped with Illumina 610K arrays as part of a GWAS of asthma [30]. As such, this dataset provided a unique opportunity to search for potential genetic modifiers of environmental risk factors. Methods used for DNA extraction, genotyping, and data quality control are described in Ferreira et al. [30]. For this study, we selected data for 26 SNPs that have previously been reported to associate with the risk of asthma or other allergic diseases in GWAS at the genome-wide significance level (*P* < 5.0 × 10^−8^, Supplementary Table S2).

### Main association analysis

Our primary outcome of interest was self-reported asthma age-of-onset, measured in years.

Age-of-onset was not normally distributed (Supplementary Fig. S1) and so we first applied a non-parametric transformation (inverse normal). Next, we used linear regression to test for individual effects of sex, study group, and family history on age-of-onset and found that sex and study were significant (*P* < 0.05) predictors of age-of-onset. For this reason, subsequent analyses were performed after adjusting for sex and study (i.e., using the residuals from the regression of sex and study on age-of-onset).

We performed linear regression to test for associations between environmental exposures and the normalized, sex- and study-adjusted age-of-onset. We used a permutation-based method to correct the observed individual association *P*-values (*P*_uncorrected_) for the number of environmental exposures tested (i.e., 17). Briefly, we (1) permuted age-of-onset values across the 1085 asthmatics; (2) tested each of the 17 exposures for association with the permuted age-of-onset values, as described above; (3) retained the lowest *P*-value recorded across the 17 exposure variables (*P*_min_); (4) repeated (1) to (3) 10,000 times; and (5) for each exposure, calculated the corrected *P*-value as: *P*_corrected_ = number of permutation datasets with *P*_min_ ≤ *P*_uncorrected_ / 10,000.

### Secondary association analyses

For environmental variables that were significantly associated with age-of-onset (*P*_corrected_ < 0.05), we performed five additional secondary analyses. First, we dichotomized age-of-onset into early childhood asthma onset (between the ages of two and six) and later onset (at or after the age of six). To evaluate the associations between individual exposures and early childhood onset, 1-df chi-square tests of independence were used. Odds ratios, standard errors, and 95% confidence intervals were calculated from the two-by-two contingency tables generated for these tests.

Second, we tested for sex-specific and study-specific effects by analyzing the normalized age-of-onset data, prior to the adjustment for sex or study. This was performed by including an interaction term between sex (or study) and the exposure in linear regression models, and testing if the regression coefficient for this interaction term was significantly different from zero.

Third, to test whether the effect of specific exposures on asthma age-of-onset was likely to be mediated through an effect on allergic sensitization, we repeated the association analysis while accounting for atopy status. This analysis was performed in a subset of 402 subjects who underwent allergy testing.

Fourth, we investigated the effect of exposure to pairs of environmental risk factors on age-of-onset. In these analyses, we considered as exposed those individuals reporting the presence of two risk factors, and as unexposed those individuals who reported the absence of both risk factors. Chi-square tests of independence were again utilized to test for association between early childhood asthma onset and the bivariate exposure.

Lastly, to identify potential genetic modifiers of environmental exposures, we tested for interactions between 26 individual SNPs and environmental exposures significantly associated with age-of-onset, using data from 318 asthmatics and the same approach described above for the analysis of sex and study-specific effects. We assumed an additive genetic model, with SNPs coded as 0, 1, or 2 copies of the minor allele. Again, we used a permutation-based correction for multiple testing, as described above.

## Results

### Description of participants

The study cohort consisted of 1085 unrelated individuals with physician-diagnosed asthma and an onset of asthma at or after two years of age (Table1). Subjects with an onset of asthma before 2 years of age were excluded to ensure that environmental exposures, occurring during the first 2 years of life, preceded asthma development. Of the 1085 individuals, 682 (63%) were tested between 1992 and 1994 (Study 1), and 403 (37%) were tested between 1995 and 1998 (Study 2).

**Table 1:** Descriptive Characteristics of the Study Population (*N* = 1085)

	Overall	Study 1	Study 2
Number of subjects	1085	682	403
Females (%)	61	65	55**
Asthma age-of-onset < 6 (%)	31	27	38***
Family history of allergic disease (%)	81	76	92***
Eczema (%)	34	32	38*
Hayfever (%)	64	55	77***
Atopy (%), *N* = 402	81	81	81
FEV_1_ < 80% predicted (%)^a^, *N* = 468	19	19	19
Mean log total serum IgE (range), *N* = 444	4.7 (0.7–8.4)	4.4 (0.7–7.6)	4.9 (1.6–8.4)**

FEV_1_, Forced expiratory volume after 1 second; IgE, Immunoglobulin E.

Significant difference in frequency or mean when compared to Study 1:

* *P* < 0.05;

** *P* < 0.005;

*** *P* < 0.0005.

a Predicted FEV_1_ was calculated based on each subject's age and sex.

This cohort is representative of individuals at a high-risk for allergic disease, with 81% reporting one or more first-degree relatives with a previous diagnosis of asthma, hay fever or eczema. Of those clinically tested, most (81%) were allergic, as defined by a positive skin-prick test to at least one common allergen. Asthma age-of-onset varied between 2 and 72 years (mean 14.6, median 10), with males reporting an earlier age-of-onset than females (mean 11.7 vs. 16.4, median 7 vs. 13.5, Kruskal–Wallis test *P* = 4 × 10^−11^). Thirty-one percent of subjects reported an early childhood onset of asthma, defined by a disease onset between the ages of 2 and 6. When compared to Study 1, asthmatics from Study 2 were more likely to have early childhood disease onset (27% vs. 38%), report a family history of allergic disease (76% vs. 92%), the co-occurrence of eczema (32% vs. 38%) and hayfever (55% vs. 77%), and have higher mean log total serum IgE levels (4.4 vs. 4.9, Table1). These differences largely reflect the different ascertainment schemes used in both studies (see Methods section for details).

### Individual environmental determinants of asthma age-of-onset

We investigated the hypothesis that environmental exposures during the first two years of life may be associated with variation in asthma age-of-onset. We tested 17 specific exposures (Supplementary Table S3) reported retrospectively by each subject or by a parent (for children under the age of 16). A number of these exposures were highly correlated with each other (Supplementary Table S4), for example, subjects living in a brick house were 3.2 times more likely to be exposed to carpet (CI 2.4–4.3, *P* = 5 × 10^−16^) than subjects living in a wood house. For this analysis, age-of-onset was normalized and adjusted for sex and study effects.

Overall, five environmental exposures under the age of two were significantly associated with variation in age-of-onset after a permutation-based correction for multiple testing (Table2). The significant associations were for living in a brick house (*P* = 3 × 10^−5^), exposure to carpet (*P* = 6 × 10^−5^), suffering a serious chest illness (*P* = 10^−4^), having a father who was a cigarette smoker (*P* = 6 × 10^−4^) and direct exposure to paternal smoking (*P* = 3 × 10^−4^). Of these significant variables, only carpet exposure was also associated with atopy status (OR 1.8, CI 1.0–3.0, *P* = 0.041). The effect of carpet exposure on asthma age-of-onset remained significant after accounting for atopy status (*P* = 0.008).

**Table 2:** Association between environmental exposures during the first 2 years of life and variation in asthma age-of-onset

Environmental variable	Sample size	*t*-Statistic	*P*-value	
			Uncorrected	Corrected^a^
**Type of house (brick or wood)**	**977**	**4.233**	**2.5 × 10**^−**5**^	**1 × 10**^**−4**^
**Carpet exposure**	**1002**	**5.152**	**6.2 × 10**^−**5**^	**5 × 10**^**−4**^
**Serious chest illness**	**1001**	**3.833**	**1.4 × 10**^−**4**^	**0.0016**
**Direct exposure to father's smoking**	**377**	**−4.148**	**2.6 × 10**^−**4**^	**0.0033**
**Father a cigarette smoker**	**1050**	**−3.430**	**6.3 × 10**^−**4**^	**0.0103**
Breastfeeding as an infant (under 6 months of age)	348	2.196	0.0287	0.3878
Location of house (major city/town or rural area)	1077	1.935	0.0533	0.5978
Otitis media	353	1.433	0.1526	1
Duration of breastfeeding	287	−1.285	0.1997	1
Dog exposure	1065	−1.204	0.2290	1
House less than 50 m from a main road	1027	−1.048	0.2950	1
Quantity of cigarettes smoked daily by mother	58	0.863	0.3119	1
Direct exposure to mother's smoking	388	−0.762	0.4460	1
Cat exposure	1063	−0.758	0.4490	1
Mother a cigarette smoker during pregnancy	390	−0.552	0.5810	1
Quantity of cigarettes smoked daily by father	137	−0.156	0.8761	1
Mother a cigarette smoker	1060	0.138	0.8900	1

a *P*-value is corrected for multiple testing by 10,000 permutations.

Statistically significant associations (*P* < 0.05) after correction for multiple testing are displayed in bold.

**Figure 1:**
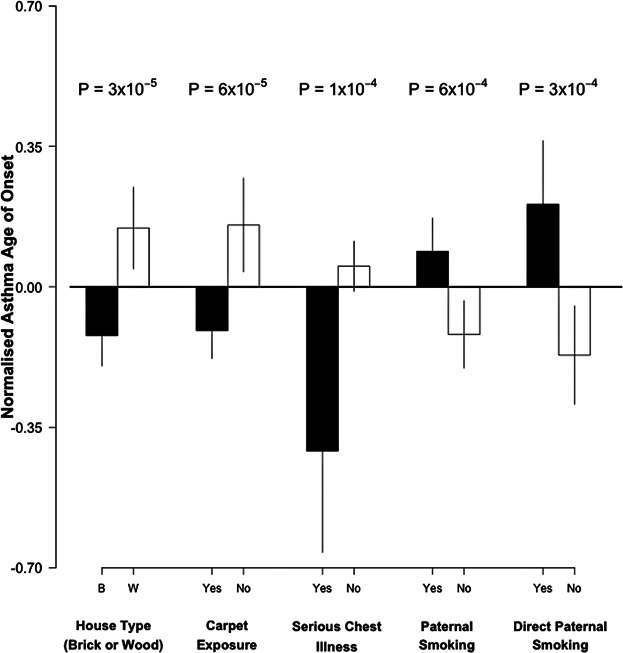
Mean asthma age-of-onset according to the exposure to five significant environmental predictors of variation in age-of-onset. Age-of-onset was normalized and adjusted for sex and study type prior to the association analysis.

Subjects living in a house with carpet had a significantly lower mean age-of-onset of asthma than subjects without this exposure (Fig. 1). When considering an age-of-onset between the ages of two and six to define early childhood onset of asthma, then asthmatics growing up in a house with carpet were 1.4-times (CI 1.1–1.9, *P* = 0.018) more likely to report early childhood onset than asthmatics without carpet at home (Table3). Carpet exposure was highly associated with living in a brick house (*P* = 5 × 10^−16^, Supplementary Table S4), and so association results for house type largely reflected those observed with carpet exposure (OR 1.4, CI 1.1–1.9, *P* = 0.014).

**Table 3:** Association between early life environmental exposures and the development of early childhood onset asthma (2–6 years old)

	Frequency of exposure (%)		OR (95% CI)	Standard error	*P*-value
	Asthma onset < 6	Asthma onset ≥ 6			
Serious chest illness	11	5	2.1 (1.3–3.5)	0.249	0.0020
Carpet exposure	71	64	1.4 (1.1–1.9)	0.147	0.0175
Brick house type	66	57	1.4 (1.1–1.9)	0.142	0.0139
Paternal smoking	43	56	0.6 (0.5–0.8)	0.133	0.0002
Direct exposure to paternal smoking	27	45	0.5 (0.3–0.7)	0.227	0.0008

OR, odds ratio; CI, confidence interval.

The occurrence of a serious chest illness (not including asthma) in the first 2 years of life was also associated with an earlier asthma age-of-onset (Fig. 1). This exposure was associated with a 2.1-fold increased risk of early childhood asthma (CI 1.3–3.5, *P* = 0.002, Table3).

On the other hand, for the correlated variables (*P* = 4 × 10^−68^, Supplementary Table S4) of paternal smoking and direct exposure to paternal smoking, the direction of effect was counter-intuitive, with subjects who had these exposures reporting a later mean age-of-onset when compared to those without these exposures (Fig. 1). For example, asthmatics who were directly exposed to paternal smoking during the first two years of life, were two times (CI 1.4–3.3, *P* = 0.0008) more likely to report developing asthma after 6 years of age (Table3).

### Sex- and study-specific effects of environmental exposures on asthma age-of-onset

For the five environmental exposures significantly associated with variation in asthma age-of-onset, we tested whether the effect was comparable between males and females, and also between the two study groups included in this analysis (Study 1 and 2). The effect for all five exposures was comparable between males and females (*P* > 0.05, Supplementary Table S5). On the other hand, although the direction of effect was consistent between the two study groups for all five exposures, two variables had stronger effects on asthma age-of-onset in Study 2 when compared to Study 1: carpet exposure and paternal smoking (Supplementary Table S6).

### Impact of multiple environmental exposures on age-of-onset

Next, we estimated the effect on age-of-onset of the combination of pairs of environmental exposures (Table4). The exposures selected for analysis were all significantly associated with age-of-onset when tested individually; of the two correlated paternal smoking variables, we selected for analysis the variable with the largest sample size (i.e., having a father who was a cigarette smoker). Of note, asthmatics who reported both carpet exposure and a serious chest illness during the first 2 years of life were 3.2 times (CI 1.7–6.0, *P* = 2 × 10^−4^) more likely to report developing early childhood asthma, between the ages of two and six, when compared to asthmatics with neither of these exposures.

**Table 4:** The combined effects of pairs of environmental exposures on asthma age-of-onset

	Paternal smoking	Serious chest illness	Brick house type
Carpet exposure	0.8 (0.6–1.3)	3.2 (1.7–6.0)***	1.7 (1.1–2.5)*
Paternal smoking	NA	1.2 (0.6–2.5)	0.9 (0.6–1.4)
Serious chest illness	NA	NA	2.2 (1.2–4.0)*

Results are presented as: odds ratio (95% confidence interval).

* *P* < 0.05;

** *P* < 0.0005.

### Genetic modifiers of individual environmental determinants of asthma-age-of onset

There is significant variation in asthma age-of-onset amongst individuals with the same environmental exposure (Supplementary Fig. S2). We hypothesized that the effect of early life environmental determinants on the asthma age-of-onset may be modified by genetic risk factors. To identify possible genetic modifiers, we tested for interactions between individual SNPs and the five significant (after correction for multiple testing) environmental determinants of asthma age-of-onset identified above, in a subset of 318 asthmatics with available GWAS data. Our focus was on individual SNPs, rather than on a polygenic risk score that combined the effects of multiple SNPs, because we were interested in identifying specific genes that protect against environmental risk factors and so that may point to new drug targets for asthma.

Twenty-six SNPs were selected due to their established association with risk of asthma or allergic disease through previous GWAS (Supplementary Table S2, *P* < 5.0 × 10^−8^). Individual SNPs were then tested for interaction with the significant environmental determinants of brick house type, carpet exposure, suffering a serious chest illness, paternal smoking, and direct exposure to paternal smoking. Overall, there were no statistically significant interactions after correcting for the 130 tests performed through permutations (Table5). The strongest interactions were observed between rs4129267 and carpet exposure (*P* = 0.028), rs9500927 and paternal smoking (*P* = 0.032) and rs10508372 and both paternal smoking (*P* = 0.012) and direct exposure to paternal smoking (*P* = 0.011).

**Table 5:** Interactions between SNPs and environmental determinants of asthma age-of-onset

Environmental determinant	SNP	Risk allele	Gene	Position	Interaction test		
					*t*-Statistic	*P*-value	
						Uncorrected	Corrected^a^
Carpet exposure	rs4129267	T	*IL6R*	1q21.3	−2.215	0.0275	0.9740
Paternal smoking	rs10508372	C	*LOC338591*	10p14	−2.523	0.0122	0.8000
	rs9500927	T	*HLA-DOA*	6p21.32	2.151	0.0322	0.9865
Direct exposure to paternal smoking	rs10508372	C	*LOC338591*	10p14	−2.564	0.0108	0.7627

a *P*-value is corrected for multiple testing by 10,000 permutations.

## Discussion

We have identified early life carpet exposure, living in a brick house type and suffering a serious chest illness as environmental predictors of early-onset asthma while paternal smoking exposure was associated with a later onset of asthma. We also found that suffering a serious chest illness concomitantly with exposure to carpet before the age of two enhanced the individual effects of these environmental risk factors on age-of-onset.

In the group of asthmatics included in this study, males were approximately two times more likely than females to report an onset of asthma between the ages of two and six. This sex difference in age-of-onset is consistent with previous studies that report a higher incidence of asthma in males prior to adolescence, with a pattern change during adolescence where asthma onset is more prevalent in females [31,32]. Despite this difference in age-of-onset between sexes, we did not find that environmental exposures with an overall association with age-of-onset had stronger effects in either sex.

The two environmental exposures most strongly associated with asthma age-of-onset in our study were the presence of carpet at home and living in a brick house type in early life. These two variables were highly associated with each other, as individuals exposed to carpet were three times more likely to live in a brick house as opposed to a wood house.

Exposure to carpet has been linked with an increased risk of asthma in case–control studies [21,33] but, to our knowledge, no studies to date had analyzed its effect on asthma age-of-onset. The mechanism explaining the association between exposure to carpet and both disease status and disease age-of-onset is likely to be increased exposure to environmental allergens, such as house dust mites. Households with carpet have been reported to have significantly higher levels of house dust mite allergens, such as Der p I and Der f 1, when compared to households with hard flooring [34,35]. Exposure to these allergens in early life is a strong risk factor for the later development of asthma [17,36–38]. Therefore, our results suggest that disease onset in children at a high-risk of developing asthma can potentially be delayed by avoiding exposure to carpet at home during the first 2 years of life.

A similar mechanism can be hypothesized for the effect of a brick house type on early onset asthma as brick cladding in houses has been associated with a high concentration of household dust mites [39]. Thus, it is possible that living in a brick house in early life leads to an increased exposure to dust mites, which then increases the risk of developing asthma in early childhood [36–38]. This is further supported by the 1.7-fold increased risk of early childhood asthma when subjects report living in a brick house with carpet, as opposed to a wood house without carpet, before the age of two. However, due to the strong association between carpet exposure and a brick house type, one of these variables may be predicting exposure to the other and the subsequent increased risk of early childhood asthma. Notably, carpet exposure but not living in a brick house was significantly associated with atopy status in this study. In addition, atopy was significantly associated with an earlier asthma age-of-onset. These associations suggest that carpet exposure increases the risk of allergic sensitization which, in turn, may precipitate the development of asthma. However, because the effect of carpet exposure on age-of-onset remained significant after adjusting for atopy status, our results suggest that this exposure influences asthma age-of-onset above and beyond its potential effect on the development of allergic sensitization.

Our findings also suggest that the occurrence of a serious chest illness under the age of two may significantly precipitate an early onset of asthma. These results are consistent with a large body of evidence linking viral [40–42] and bacterial [43,44] respiratory infections in early life with an increased risk of early-onset asthma. It currently remains uncertain if a causal relationship exists between respiratory infections and asthma or if these infections identify individuals with a predisposing factor for asthma development [45–48].

In this study, environmental tobacco smoke (ETS) exposure was assessed through maternal and paternal questionnaire items. There were no significant associations between prenatal and postnatal maternal smoking, despite these exposures having previously been reported to be a risk factor for asthma [49–51]. On the other hand, we found that asthmatics reporting paternal smoking or direct exposure to paternal smoking during the first two years of life were more likely to develop asthma later in life. This direction of effect is counter-intuitive, but consistent with two previous studies that found that ETS exposure in early life was a stronger risk factor for later as opposed to early onset asthma [21,52]. Further support is apparent in studies correlating childhood ETS exposure with adult onset asthma [51,53,54]. One possible explanation for this observation, as proposed partly by Jaakkola et al. [52], is that chronic inflammation of the airways caused by ETS exposure may trigger late-onset asthma in individuals who otherwise would not have developed asthma. Nonetheless, there are conflicting studies that reported an association between ETS exposure and an earlier asthma onset [55,56]. Therefore, further studies are required to elucidate the effects of both prenatal and postnatal smoking exposure on asthma age-of-onset.

We also found that if environmental exposures co-occurred, the risk of developing asthma in early childhood was significantly increased when compared to the individual exposures. For example, subjects who reported both carpet exposure and suffering from a serious chest illness during their first 2 years of life were three times more likely to have an early childhood onset of asthma, when compared to subjects without both of these exposures. Support for this finding can be found in recent studies reporting that children at the highest risk of developing asthma are those who, before 2 years of age, suffered from viral respiratory infections and allergic sensitization [47,57]. It has also been postulated, based on results from a mouse model, that viral respiratory infections prime immunological pathways and lead to an increased responsiveness to house dust mite allergens, subsequent allergic sensitization and asthma [58]. On the other hand, there is evidence for a causal relationship between allergic sensitization and an increased susceptibility to viral infections leading to asthma [59,60]. Thus, our findings are consistent with a rapid progression towards asthma development following these two exposures in early life.

Lastly, we explored the hypothesis that genetic risk factors for asthma modify the effect of early life environmental determinants on asthma age-of-onset. However, overall, we found no significant interactions between the environmental exposures and the 26 SNPs tested. There have been reports of interactions between genetic susceptibility variants and environmental risk factors for asthma development [25,26,61]. For example, 17q21 genetic variants have been found to interact with early life ETS exposure and early-onset asthma [27,28]. The absence of significant genetic interactions with the environmental determinants tested in our study may reflect a true negative result but, given the relative small sample size of 318 subjects in this analysis, it may also represent a false negative finding. Because identification of genes that protect against environmental risk factors may point to new drug targets for asthma, studies that explore potential genetic modifiers for both of these exposures with larger sample sizes and/or by testing more SNPs, are justified.

This study suffered from additional limitations, which need to be considered when interpreting the reported associations. First, information about environmental exposures was reported retrospectively by participants, an approach that is affected by recollection bias. This limitation could be addressed through the analysis of data collected by longitudinal studies of children at a high risk of developing asthma, as these studies provide a more precise characterization of the types and length of exposures encountered in early life. Second, to increase sample size, we combined data collected as part of two studies, which used very comparable epidemiological questionnaires to measure environmental exposures but that employed different strategies to ascertain asthmatics. As a result, Study 2 had a greater enrichment of asthmatics with eczema, hay fever and a family history of allergic disease than Study 1. To some extent, this difference may underlie the consistently larger effect observed in Study 2 for the five significant environmental exposures.

In summary, we found that exposure to carpet at home and suffering from a serious chest illness during early life can lead to an earlier onset of asthma in a high-risk study cohort. As such, avoiding these exposures in high-risk individuals may delay the onset of asthma and, in this way, reduce the burden of disease.

## Supporting Information

**Figure S1.** Asthma age-of-onset distribution. The range of age-of-onset was from 2 to 72 years of age (*N* = 1085). **Figure S2.** Variation in asthma age-of-onset within significant environmental exposure groups. **Table S1.** Asthma and allergy questionnaire items selected for analysis. **Table S2.** Single nucleotide polymorphisms selected for gene-by-environment interaction. **Table S3.** Frequency of individual and pairs of exposures in the study cohort. **Table S4.** Associations (*P*-value) between pairs of environmental exposures. **Table S5.** Analysis of heterogeneity of effect between sexes for early life environmental exposures on asthma age-of-onset. **Table S6.** Analysis of heterogeneity of effect between the two study types for early life environmental exposures on asthma age-of-onset.
