# Supplementary material for: Early life environmental predictors of asthma age-of-onset
Source: Immun Inflamm Dis. 2014 Jul 26;2(3):141–51. doi: 10.1002/iid3.27 (PMC4257759; doi:10.1002/iid3.27)

**Early life environmental predictors of asthma age-of-onset**

Olivia R Ferry, David L Duffy and Manuel AR Ferreira

Supporting Information

**Supporting Figure 1:** Asthma age-of-onset distribution. The range of age-of-onset was from two to seventy two years of age (N=1,085).

**
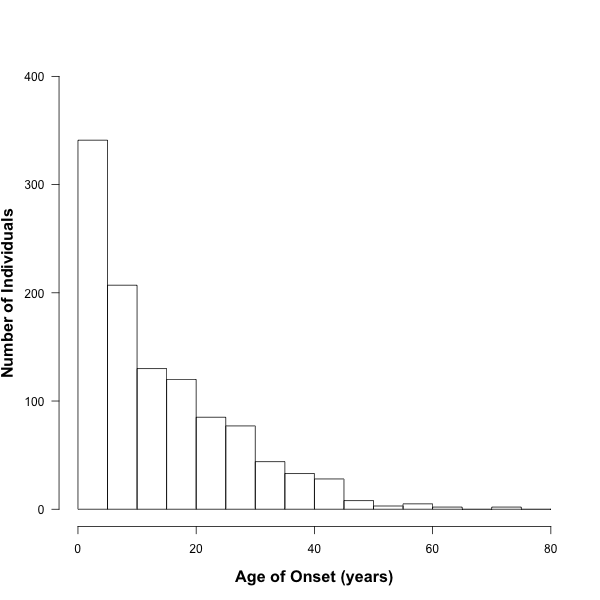
**

**Supporting Table 1**: Asthma and allergy questionnaire items selected for analysis.

| Question | Responses |
| --- | --- |
| ^a^ BEFORE THE AGE OF 5 YEARS did you have a serious chest illness or illnesses (not including asthma)?  What was the age that the serious chest illness FIRST occurred? | Yes, no  (years) |
| Did you have a lot of otitis media (ear infections) when you were under the age of two? | Yes, no, unsure |
| Did you have contact with a cat at home when you were under the age of two? | Yes, no, unsure |
| Did you have contact with a dog at home when you were under the age of two? | Yes, no, unsure |
| ^b^ What type of area did you live in when you were under the age of two? (for most of this time) | In a major city, in a town, in a rural area, unsure |
| Did you live within 50 metres of a main road when you were under the age of two? | Yes, no, unsure |
| ^c^ What kind of a home did you live in when you were under the age of two? (for most of this time) | A brick house or unit, a wooden house or unit, other type of accommodation, unsure |
| Was your home carpeted when you were under the age of two? | Yes, no, unsure |
| Was your mother a smoker when she was pregnant with you? | Yes, no, unsure |
| Was your mother a smoker when you were under the age of two? | Yes, no, unsure |
| Did your mother smoke around you when you were under the age of two? | Yes, no, unsure |
| ^d^ How many cigarettes on average did your mother smoke per day when you were under the age of two? | 10 or less, 11 to 20, more than 20, unsure |
| Was your father a smoker when you were under the age of two? | Yes, no, unsure |
| Did your father smoke around you when you were under the age of two? | Yes, no, unsure |
| ^d^ How many cigarettes on average did your father smoke per day when you were under the age of two? | 10 or less, 11 to 20, more than 20, unsure |
| ^e^ How were you fed as a baby up until the age of 6 months? | Only breastfed, mainly breastfed, mainly formula, only formula, other/unsure |
| ^f^ If you were breastfed at all, how long was this for? | Less than 1 month, 1 – 3 months, 3 – 6 months, more than 6 months |

For all questions, responses of ‘unsure’ were considered as missing.

**^a^** These two questions were used to classify subjects as suffering a serious chest illness under the age of two.

**^b^** Responses grouped into major city/town (in a major city or in a town) and rural area

**^c^** Subjects who responded ‘other’ were considered as missing.

**^d^** Responses grouped into 10 or less and 11 or more (11 – 20 or more than 20)

**^e^** Responses grouped into breastfed (only or mainly breastfed) and formula fed (only or mainly formula).

**^f^** Responses grouped into less than 3 months (less than 1 month or 1 – 3 months) and greater than 3 months (3 – 6 months or more than 6 months).

**Supporting Table 2**: Single nucleotide polymorphisms selected for gene-by-environment interaction analysis.

| SNP | Risk Allele | Gene | Position | Reference | Odds Ratio | P-value |
| --- | --- | --- | --- | --- | --- | --- |
| rs4129267 | T | *IL6R* | 1q21.3 | Ferreira MA | 1.09 | 2.0e-08 |
| rs3771180 | G | *IL1RL1* | 2q12.1 | Torgerson DG | NA | 2.0e-15 |
| rs13408661 | G | *IL1RL1, IL18R1* | 2q12.1 | Ramasamy A | 1.23 | 1.0e-09 |
| rs3771166 | G | *IL18R1* | 2q12.1 | Moffatt MF | 1.15 | 3.0e-09 |
| rs7686660 | T | *LOC729675* | 4q31.21 | Hirota T | 1.16 | 2.0e-12 |
| rs1837253 | C | *TSLP* | 5q22.1 | Hirota T | 1.17 | 1.0e-16 |
| rs404860 | A | *NOTCH4* | 6p21.32 | Hirota T | 1.21 | 4.0e-23 |
| rs204993 | A | *PBX2* | 6p21.32 | Hirota T | 1.17 | 2.0e-15 |
| rs3129943 | T | *C6orf10* | 6p21.32 | Hirota T | 1.17 | 3.0e-15 |
| rs3117098 | G | *BTNL2* | 6p21.32 | Hirota T | 1.16 | 5.0e-12 |
| rs9268516 | T | *BTNL2, HLA---DRA* | 6p21.32 | Ramasamy A | 1.15 | 1.0e-08 |
| rs3129890 | T | *HLA---DRA* | 6p21.32 | Hirota T | 1.15 | 5.0e-13 |
| rs7775228 | A | *HLA---DQB1* | 6p21.32 | Hirota T | 1.17 | 5.0e-15 |
| rs9275698 | T | *HLA---DQA2* | 6p21.32 | Hirota T | 1.18 | 5.0e-12 |
| rs9500927 | T | *HLA-DOA* | 6p21.32 | Hirota T | 1.13 | 4.0e-09 |
| rs987870 | C | *HLA, DPB1* | 6p21.32 | Noguchi E | 1.40 | 2.0e-10 |
| rs3019885 | G | *SLC30A8* | 8q24.11 | Noguchi E | 1.34 | 5.0e-13 |
| rs1342326 | C | *IL33* | 9p24.1 | Moffatt MF | 1.20 | 9.0e-10 |
| rs2381416 | C | *IL33* | 9p24.1 | Torgerson DG | NA | 2.0e-12 |
| rs10508372 | C | *LOC338591* | 10p14 | Hirota T | 1.16 | 2.0e-15 |
| rs7130588 | G | *LRRC32* | 11q13.5 | Ferreira MA | 1.13 | 2.0e-08 |
| rs2069408 | C | *CDK2* | 12q13.2 | Hirota T | 1.15 | 1.0e-10 |
| rs1701704 | G | *IKZF4* | 12q13.2 | Hirota T | 1.19 | 2.0e-13 |
| rs744910 | G | *SMAD3* | 15q22.33 | Moffatt MF | 1.12 | 4.0e-09 |
| rs3894194 | A | *GSDMA* | 17q21.2 | Moffatt MF | 1.17 | 5.0e-09 |
| rs2284033 | G | *IL2RB* | 22q12.3 | Moffatt MF | 1.12 | 1.0e-08 |

**Supporting Table 3**: Frequency of individual and pairs of exposures in the study cohort.

|  | Overall (N=1,085) | | Study 1 (N=682) | | Study 2 (N=403) | |
| --- | --- | --- | --- | --- | --- | --- |
|  | N with data | % with exposure | N with data | % with exposure | N with data | % with exposure |
| *Individual exposures* | | | | | | |
| Carpet exposure | 1002 | 66 | 634 | 62 | 368 | 73** |
| Living in a brick house  (vs. wood house) | 977 | 60 | 618 | 57 | 359 | 64* |
| Serious chest illness | 1001 | 7 | 669 | 6 | 332 | 8 |
| Father a cigarette smoker | 1050 | 52 | 661 | 56 | 389 | 43*** |
| Direct exposure to father’s smoking | 377 | 38 | 0 | NA | 377 | 38 |
| Otitis media | 353 | 21 | 0 | NA | 353 | 21 |
| Mother a cigarette smoker | 1060 | 24 | 670 | 28 | 390 | 17*** |
| Direct exposure to mother’s smoking | 388 | 14 | 0 | NA | 388 | 14 |
| Mother a cigarette smoker during pregnancy | 390 | 13 | 0 | NA | 390 | 13 |
| <11 cigarettes smoked daily by mother (vs. ≥ 11) | 58 | 45 | 0 | NA | 58 | 45 |
| <11 cigarettes smoked daily by father (vs. ≥ 11) | 137 | 37 | 0 | NA | 137 | 37 |
| Living in a major city or town  (vs. rural area) | 1077 | 79 | 677 | 77 | 400 | 82 |
| House less than 50 metres  from a main road | 1027 | 39 | 636 | 44 | 391 | 32*** |
| Cat exposure | 1063 | 49 | 673 | 55 | 390 | 38*** |
| Dog exposure | 1065 | 60 | 673 | 68 | 392 | 46*** |
| Mostly breastfed as an infant  (vs. mostly formula) | 348 | 62 | 0 | NA | 348 | 62 |
| Breastfed for < 3 months as an infant  (vs. ≥ 3months) | 287 | 36 | 0 | NA | 287 | 36 |
| *Pairs of exposures* | | | | | | |
| Carpet and paternal smoking | 449 | 69 | 316 | 68 | 133 | 72 |
| Carpet and serious chest illness | 344 | 14 | 248 | 10 | 96 | 23** |
| Carpet and brick house type | 615 | 71 | 392 | 66 | 223 | 80*** |
| Serious chest illness and  brick house type | 397 | 13 | 285 | 11 | 112 | 17 |
| Serious chest illness and  paternal smoking | 471 | 8 | 288 | 8 | 183 | 7 |
| Paternal smoking and  brick house type | 451 | 62 | 304 | 64 | 147 | 59 |

* Significant difference in frequency when compared to Study 1: P<0.05, ** P<0.005, *** P<0.0005

**Supporting Table 4:** Associations (P-value) between pairs of environmental exposures.

|  | Chest Illnesses | Otitis Media | Cat Exposure | Dog Exposure | Location  of House | House 50m from a road | House type | Carpet Exposure |
| --- | --- | --- | --- | --- | --- | --- | --- | --- |
| Otitis Media | 0.0031 | NA | NA | NA | NA | NA | NA | NA |
| Cat Exposure | 1 | 0.4402 | NA | NA | NA | NA | NA | NA |
| Dog Exposure | 0.1818 | 0.8285 | **3x10^-12^** | NA | NA | NA | NA | NA |
| Location of House | 0.4361 | 0.2322 | **7x10^-9^** | **4x10^-12^** | NA | NA | NA | NA |
| House 50m from a road | 0.9995 | 0.0132 | 0.7392 | 0.2649 | **6x10^-9^** | NA | NA | NA |
| House type | 0.0043 | 0.5434 | **1x10^-4^** | 0.0022 | **2x10^-15^** | 0.3734 | NA | NA |
| Carpet Exposure | 0.3713 | 0.5531 | 0.1778 | 0.4615 | **6x10^-11^** | 0.0049 | **5x10^-16^** | NA |
| Smoking in pregnancy | 0.6980 | 0.0555 | 0.0028 | 0.1021 | 0.3671 | 0.4035 | 1 | 0.7424 |
| Mother a smoker | 1 | 0.4524 | 0.0126 | 0.0056 | 0.0500 | 0.0554 | 0.1442 | 0.0438 |
| Direct exposure to maternal smoking | 0.6138 | 0.0685 | 0.0024 | 0.2153 | 0.6754 | 0.6473 | 0.5685 | 0.2480 |
| Maternal quantity of cigarettes | 0.2881 | 0.3140 | 0.2035 | 0.3210 | 0.4029 | 0.3231 | 0.4826 | 0.3502 |
| Father a smoker | 0.9533 | 0.1507 | 0.0150 | 0.0040 | 0.5599 | 0.2739 | 0.0596 | 0.0040 |
| Direct exposure to paternal smoking | 0.8442 | 0.1946 | 0.9329 | 0.4327 | 0.4024 | 0.8765 | 0.0191 | **0.0004** |
| Paternal quantity of cigarettes | 0.9017 | 0.3930 | 0.4985 | 0.5538 | 1 | 0.2814 | 0.5175 | 0.2191 |
| Feeding as an infant | 0.7711 | 0.0839 | 0.7613 | 0.0383 | 0.1356 | 0.8520 | 0.6508 | 1 |
| Breastfeeding duration | 0.0858 | 0.4650 | 0.3027 | 0.0011 | 0.0127 | 0.7396 | 0.1376 | 0.6379 |

P-value ≤ 0.0004 was used as the threshold of significance to account for multiple testing.

Significant associations are displayed in bold.

|  | Smoking in pregnancy | | Mother a smoker | Direct exposure to maternal smoking | Maternal quantity of cigarettes | Father a smoker | Direct exposure to paternal smoking | Paternal quantity of cigarettes | Feeding as an infant |
| --- | --- | --- | --- | --- | --- | --- | --- | --- | --- |
| Mother a smoker | **3x10^-56^** | | NA | NA | NA | NA | NA | NA | NA |
| Direct exposure to maternal smoking | **5x10^-56^** | **1x10^-70^** | | NA | NA | NA | NA | NA | NA |
| Maternal quantity of cigarettes | 0.0970 | | 0.2654 | 0.3622 | NA | NA | NA | NA | NA |
| Father a smoker | **3x10^-5^** | | **6x10^-19^** | **3x10^-5^** | 0.8181 | NA | NA | NA | NA |
| Direct exposure to paternal smoking | **8x10^-6^** | | **1x10^-5^** | **3x10^-6^** | 0.7588 | **4x10^-68^** | NA | NA | NA |
| Paternal quantity of cigarettes | 0.0273 | | 0.0036 | 0.0068 | 0.3676 | 0.0907 | 0.0610 | NA | NA |
| Feeding as an infant | 0.0088 | | 0.0468 | 0.1062 | 0.1216 | 0.0293 | 0.0033 | 0.4630 | NA |
| Breastfeeding duration | 0.0225 | | 0.1524 | 0.2483 | 0.2250 | 0.0641 | 0.0267 | 1 | **5x10^-40^** |

**Supporting Table 4 continued:** Associations (P-value) between pairs of environmental exposures.

P-value ≤ 0.0004 was used as the threshold of significance to account for multiple testing.

Significant associations are displayed in bold.

**Supporting Table 5**: Analysis of heterogeneity of effect between sexes for early life environmental exposures on asthma age-of-onset.

| Environmental Exposure | Male β Regression Coefficient (SE) | Female β Regression Coefficient (SE) | P-value for heterogeneity |
| --- | --- | --- | --- |
| Brick house type | 0.156 (0.101) | 0.337 (0.082) | 0.1687 |
| Carpet exposure | 0.374 (0.105) | 0.204 (0.084) | 0.2161 |
| Serious chest illness | 0.251 (0.199) | 0.565 (0.151) | 0.2170 |
| Paternal smoking | -0.134 (0.092) | -0.247 (0.080) | 0.3639 |
| Direct exposure to paternal smoking | -0.270 (0.162) | -0.455 (0.133) | 0.3743 |

SE, standard error

**Supporting Table 6**: Analysis of heterogeneity of effect between the two study types for early life environmental exposures on asthma age-of-onset.

| Environmental Exposure | Study 1, β Regression Coefficient (SE)  N=682 | Study 2, β Regression Coefficient (SE)  N=403 | P-value for heterogeneity |
| --- | --- | --- | --- |
| Carpet exposure | 0.106 (0.081) | 0.574 (0.110) | 0.0008 |
| Paternal smoking | -0.101 (0.077) | -0.399 (0.098) | 0.0181 |
| Serious chest illness | 0.412 (0.155) | 0.550 (0.190) | 0.5754 |
| Brick house type | 0.220 (0.081) | 0.358 (0.103) | 0.2982 |

SE, standard error

Direct exposure to paternal smoking was not recorded in study 1 and so this variable was not included in this analysis.

**
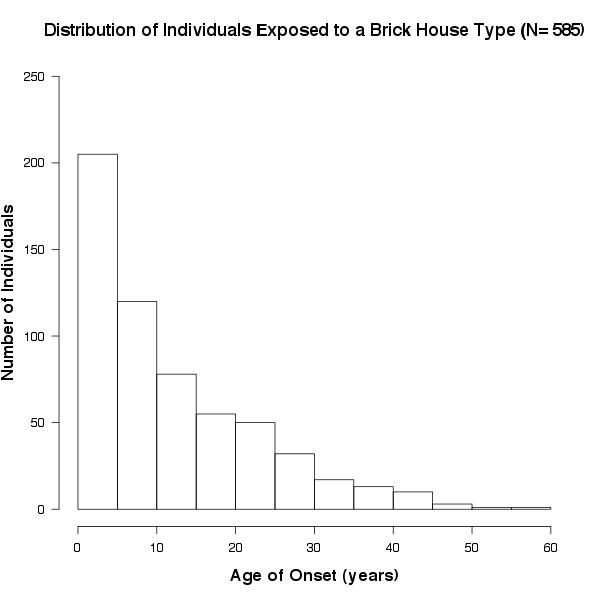
Supporting Figure 2**: Variation in asthma age-of-onset within significant environmental exposure groups.

**
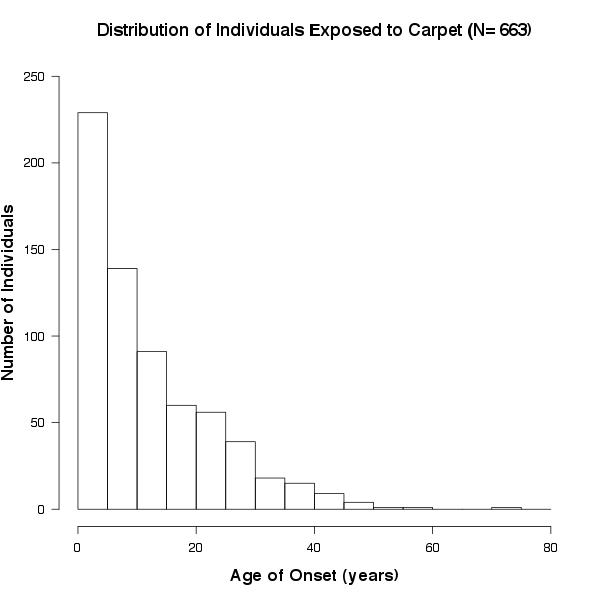
**


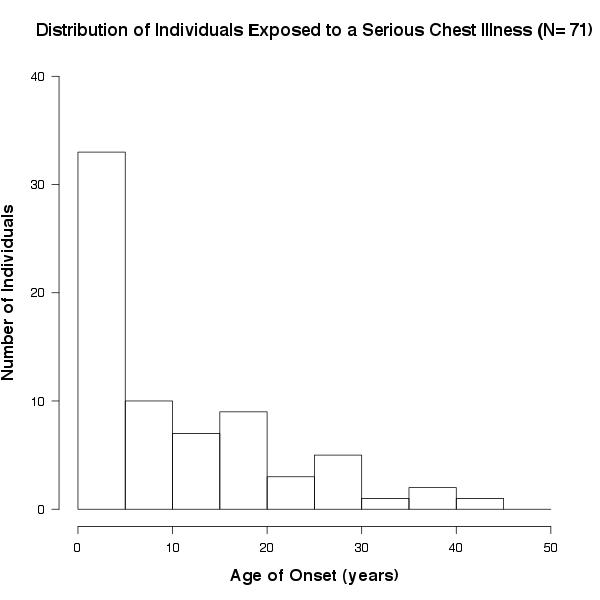

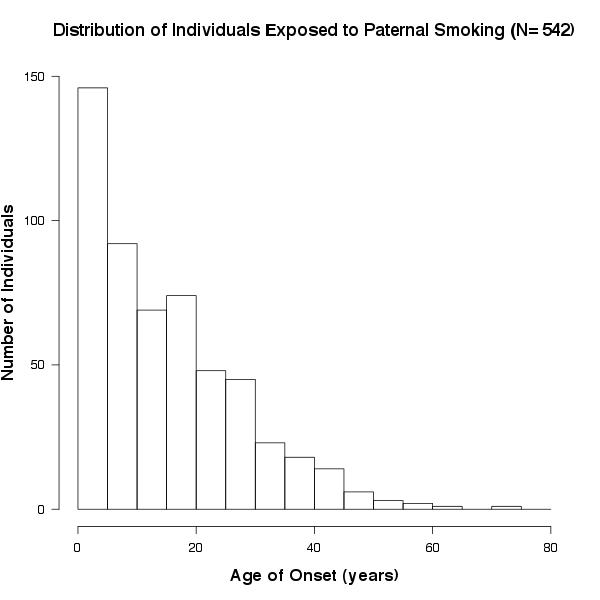

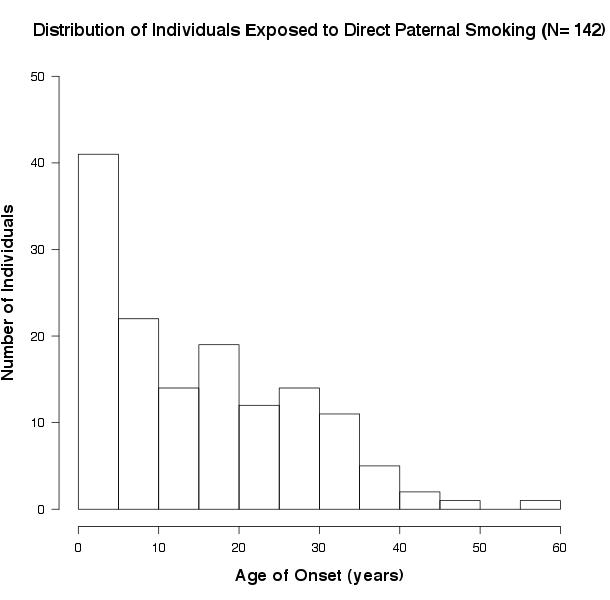

Supplement: Supplementary file 1 — Figure S1. Asthma age-of-onset distribution. The range of age-of-onset was from 2 to 72 years of age (N = 1085). Figure S2. Variation in asthma age-of-onset within significant environmental exposure groups. Table S1. Asthma and allergy questionnaire items selected for analysis. Table S2. Single nucleotide polymorphisms selected for gene-by-environment interaction. Table S3. Frequency of individual and pairs of exposures in the study cohort. Table S4. Associations (P-value) between pairs of environmental exposures. Table S5. Analysis of heterogeneity of effect between sexes for early life environmental exposures on asthma age-of-onset. Table S6. Analysis of heterogeneity of effect between the two study types for early life environmental exposures on asthma age-of-onset. [file iid30002-0141-sd1.docx]
